# Supplementary material for: Benchmarking and optimization of methods for the detection of identity-by-descent in high-recombining Plasmodium falciparum genomes
Source: eLife. 2025 Aug 19;14:RP101924. doi: 10.7554/eLife.101924 (PMC12364482; doi:10.7554/eLife.101924)

Supplementary File 2

Benchmarking and Optimization of Methods for the Detection of Identity-By-Descent in High-Recombining *Plasmodium falciparum* Genomes

Guo *et al.*

Supplementary Tables

Table S1. The default and optimized values for parameters used in inferring IBD segments via different callers. A link to the source code and a citation of the corresponding article are provided for each IBD caller. For `hap-IBD`, `Refined IBD`, and `hmmIBD`, the parameters are used on the command line except that the parameter rec_rate of `hmmIBD` needs to be specified in the source code file `hmmIBD`.c. For `phased IBD` and `isoRelate`, the parameters are specified within a Python or R script. The details of how the parameters are specified can be found in the scripts on GitHub (`https://github.com/bguo068/bmibdcaller_simulations/tree/main/bin`). Note that `mincm` and `minmaf` are values shared across IBD callers to allow fair comparisons.

IBD caller	Program parameter	Default value	Optimized/used value
`hap-IBD`<br>• version: 1.0 23Apr20.f1a<br>• Browning *et al.* 2020	min-output	2.0	mincm *
	min-seed	2.0	mincm
	min-extend	1.0	1.0
	max-gap	1000	1000
	min-markers	100	70
`hmmIBD`<br>• github.com/glipsnort/hmmIBD<br>• Commit: a2f796e<br>• Schaffner *et al.* 2018	rec_rate (in hmmIBD.c)	7.4×10^{-7}	6.67×10^{-7}
	m	5	5
	n	no limit	100
`isoRelate`<br>• github.com/bahlolab/isoRelate<br>• Commit: 109ee47<br>• Henden *et al.* 2018	isolate.max.missing	0.1	imputed
	snp.max.missing	0.1	imputed
	maf	0.01	0.1
	minimum.length.bp	50,000	mincm $\times$ 15,000
	minsnp	20	20
`Refined IBD`<br>• version: 17Jan20.102<br>• Browning *et al.* 2013	length	1.5	mincm
	lod	3.0	1.6
	scale	data-dep	data-dep
	window	40	40
	trim	0.15	0.15
`phased IBD`<br>• github.com/23andMe/phasedibd<br>• Commit: 9a7b949<br>• Freyman *et al.* 2021	template	$\begin{bmatrix} 1 & 0 & 1 & 0 \\ 0 & 1 & 0 & 1 \\ 1 & 1 & 0 & 0 \\ 0 & 0 & 1 & 1 \\ 1 & 0 & 0 & 1 \\ 0 & 1 & 1 & 0 \end{bmatrix}$	$\begin{bmatrix} 0 & 1 & 1 & 1 \\ 1 & 0 & 1 & 1 \\ 1 & 1 & 0 & 1 \\ 1 & 1 & 1 & 0 \end{bmatrix}$
	L_m	300	80
	L_f	3.0	mincm *

Table S2. Grid/Line search to optimize IBD caller-specific parameters. Column 2 lists the optimized parameters (other parameters are not explored). Column 3 shows the tested values for each parameter. Some parameters are optimized via two steps, such as coarse search (coarse) and fine-tuning (fine-tune). Column 4 provides comments on the impact of the values on IBD accuracy (measured as false positive rates (FP) and false negative rates (FN)).

IBD caller	Program parameter	Tested values	Comment
`hap-IBD`	max-gap	(3, 30, 100, 300, 1000)	little to no effect
	min-marker	coarse: (3, 10, 30, 100)<br>finetune:(30, 40, 50, 60, 70, 80, 100)	optimal around 70
`hmmIBD`	m	(2, 5, 10)	little to no effect
	n	(10, 30, 100, 300, Inf)	little to no effect
	min-maf	(0.001, 0.01)	little to no effect
`isoRelate`	min-maf	(0.01, 0.03, 0.1)	0.1 reduces FN for longer IBD
	min-snp	(1, 3, 10, 15, 20, 40, 80, 160)	$\leq$ 40 reduces FN
`Refined IBD`	min-maf	(0.01, 0.1)	little to no effect
	lod	(1.1, 1.2, 1.4, 1.6, 1.8, 2, 3, 4, 8)	lowest FN at 1.6
	window	(20, 40)	little to no effect
	trim	(0.01, 0.02, 0.05, 0.08, 0.10, 0.12, 0.15)	little to no effect
`phased IBD`	template	tolerate 1 or 2 mismatch in every 4 SNPs	optimal to tolerate 1 mismatch
	L_m	(50, 80, 90, 100, 110, 130, 150, 200, 250, 300)	optimal around 80
	min-maf	(0.001, 0.01, 0.1)	optimal around 0.01

Table S3. Isolates in the "multi-population" data set used in empirical validation. Rows are counts of isolates from different locations ("Population" labels from MalariaGEN *Pf*7 meta information table).

Population	2012	2013	Total
AF-C	66	37	103
AF-E	58	139	197
AF-W	27	273	300
AS-SE-E	75	25	100
AS-SE-W	85	59	144
OC-NG	17	40	57
Total	328	573	901

Table S4. Isolates in the "single-population" data set for "AS-SE-E" population used in empirical validation. Rows are counts of isolates from different locations ("Country" labels from MalariaGEN *Pf*7 meta information table); columns are counts of isolates collected in a given year.

Country	2010	2011	2012	Total
Cambodia	61	82	43	186
Laos	27	27	15	69
Thailand	0	0	1	1
Vietnam	28	44	16	88
Total	116	153	75	344

Table S5. Isolates in the "single-population" data set for "AF-W-Ghana" population used in empirical validation. Rows are counts of isolates from different locations ("admin level 1" labels from MalariaGEN *Pf*7 meta information table); columns are counts of isolates collected in a given year.

Admin level 1	2016	2017	2018	Total
Eastern	0	0	6	6
Greater Accra	14	13	57	84
Upper East	63	199	212	474
Volta	21	0	0	21
Total	98	212	275	585

Supplementary Data

Data S2. Detailed IBD-level benchmarking results shown in heatmaps. Each panel (a-r) represents the FN/FP rates for a specific combination of IBD callers (`hap-IBD`, `hmmIBD`, `isoRelate`, `phased IBD`, and `Refined IBD`), demographic models (single-population model, multiple-population model, and UK human demographic model), and recombination rates (human *versus* *Pf*) as indicated in the text above the panel. Note that benchmarking for genomes with human recombination rates was not performed for `hmmIBD` and `isoRelate` as they did not scale well for large genome sizes (in base pairs). For each heatmap, the searched parameters and their values are indicated as the x and y labels and tick labels, respectively; the labels in grey background on the top indicate the IBD length bin that was used to calculate FN/FP rates (labels in grey on the left); the bold labels in grey at the bottom show either different groups (e.g., coarse search or fine-tune) or the third parameter searched (such as min-maf=0.01 and min-maf=0.00).

a, `hap-IBD`, single population model, *Pf* recombination rate.

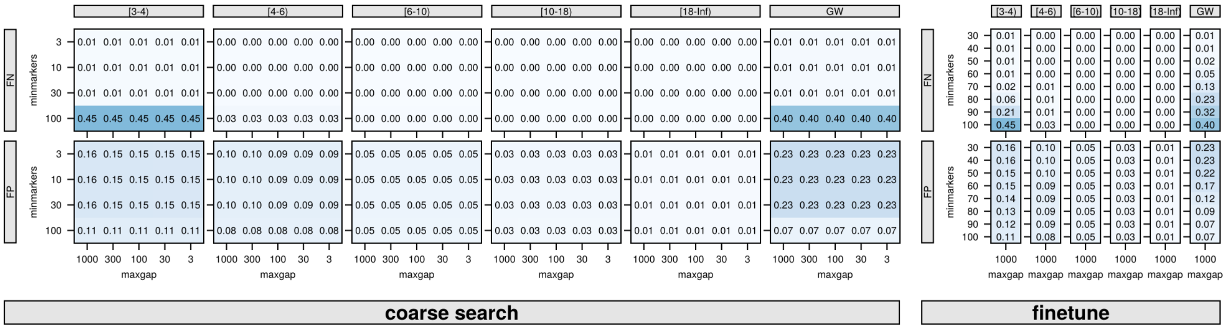

b, `hap-IBD`, multiple population model, *Pf* recombination rate.

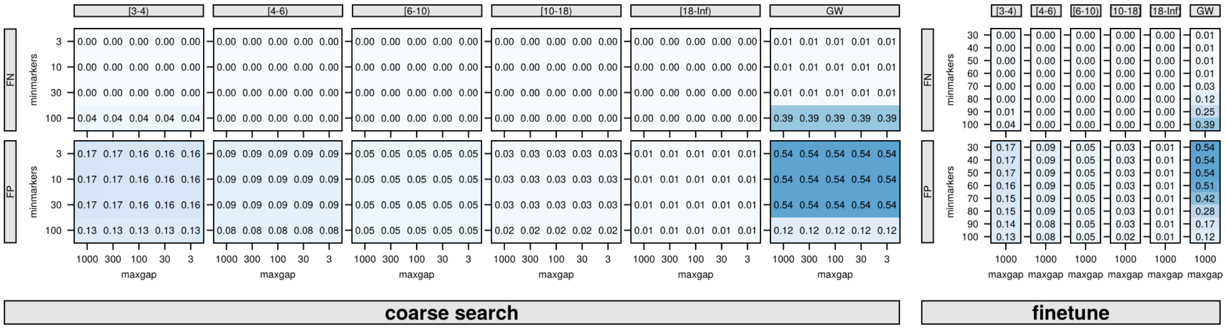

c, `hap-IBD`, UK human population model, Human recombination rate.

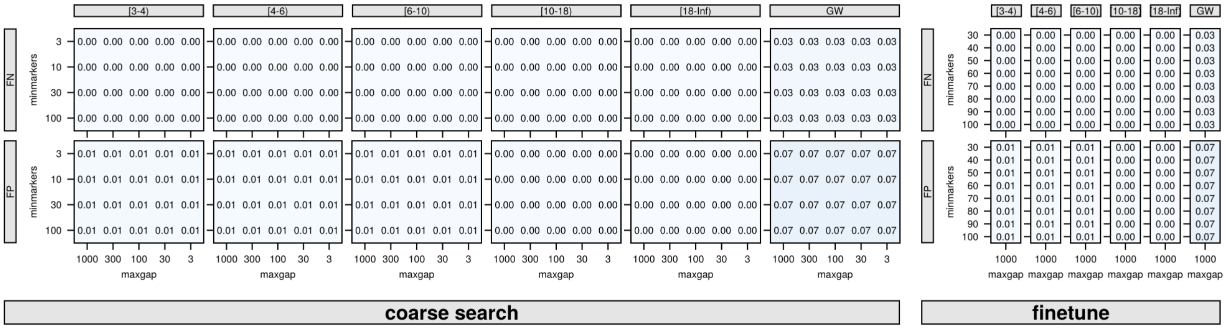

d, `hap-IBD`, UK human population model, *Pf* recombination rate.

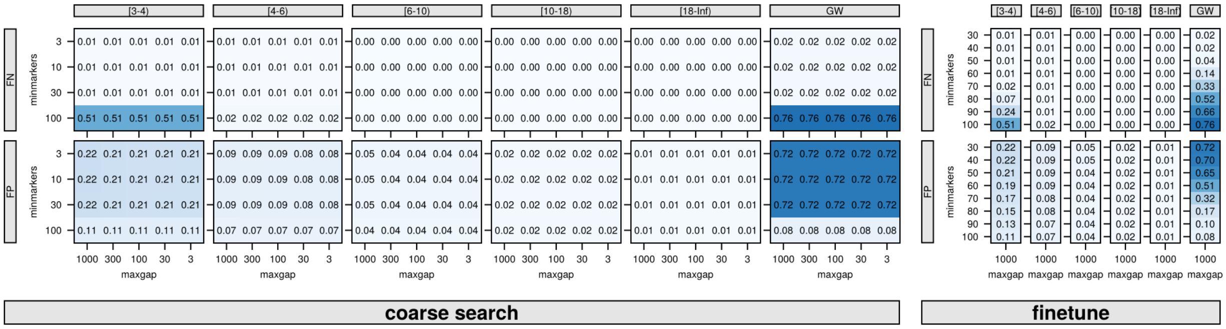

e, `hmmIBD`, single population model, *Pf* recombination rate.

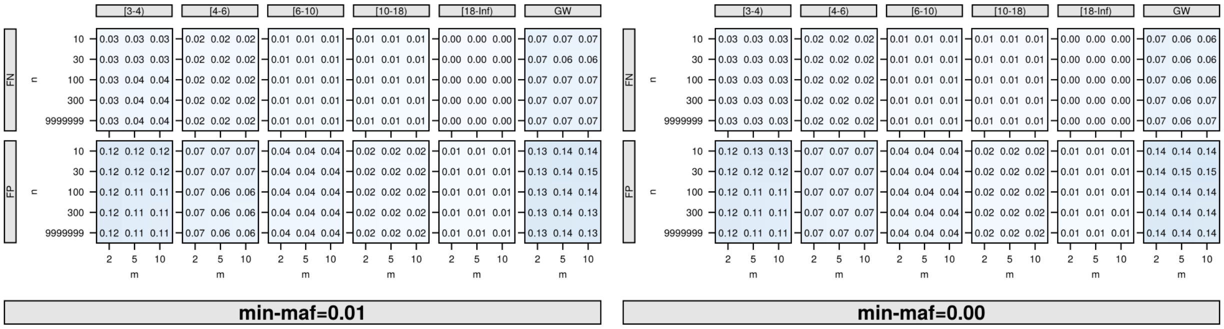

f, `hmmIBD`, multiple population model, *Pf* recombination rate.

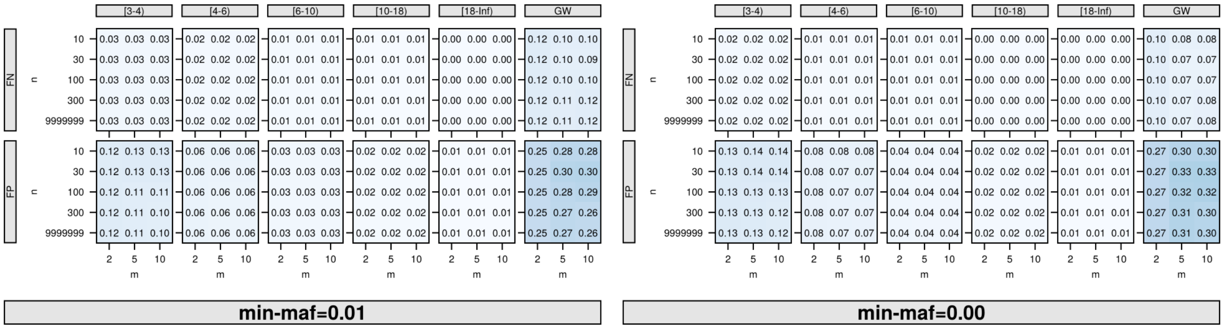

g, `hmmIBD`, UK human population model, *Pf* recombination rate.

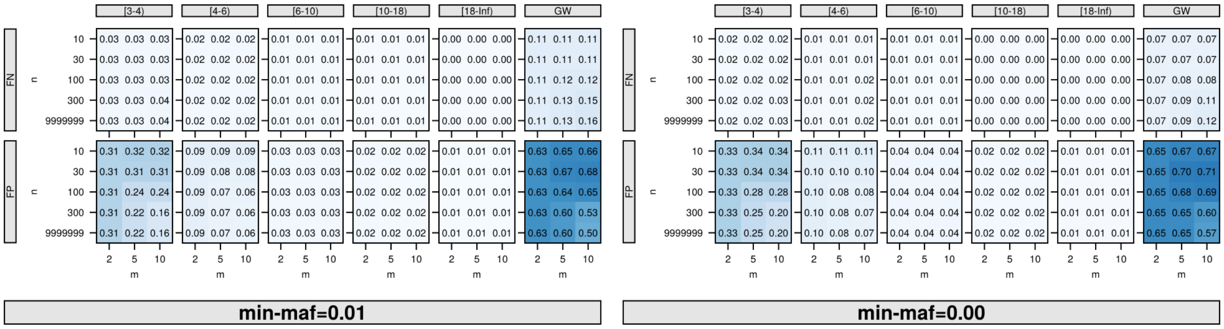

h, `isoRelate`, single population model, *Pf* recombination rate.

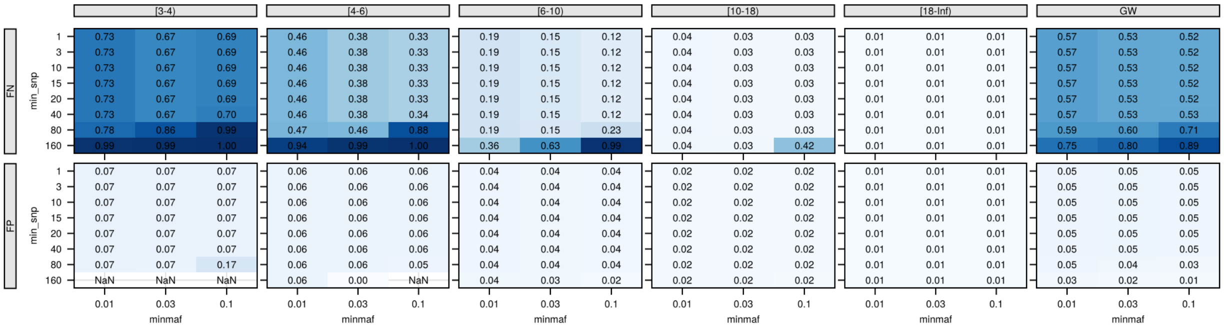

i, `isoRelate`, multiple population model, *Pf* recombination rate.

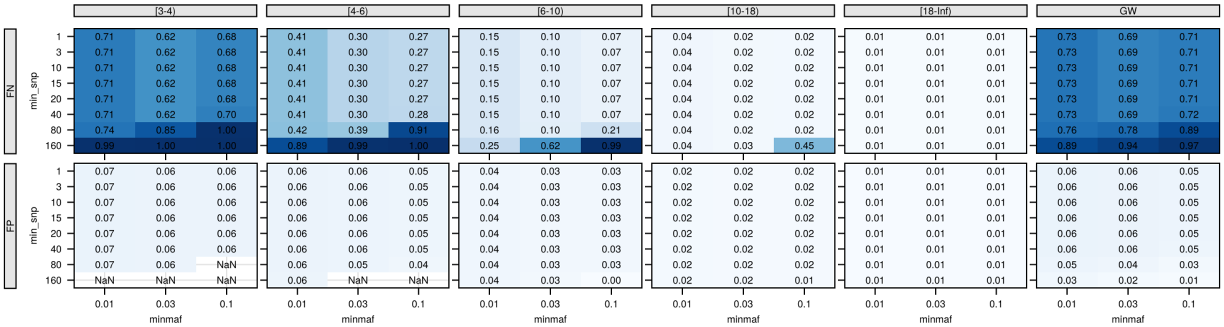

j, `isoRelate`, UK human population model, *Pf* recombination rate.

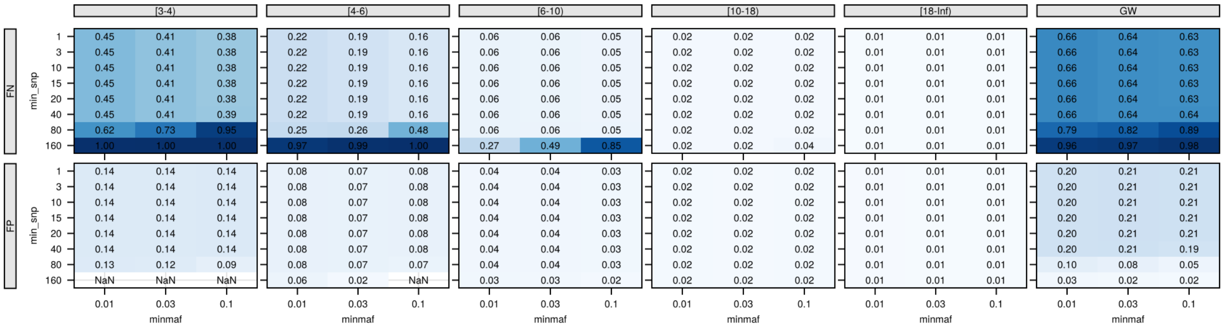

k, `Refined IBD`, single population model, *Pf* recombination rate.

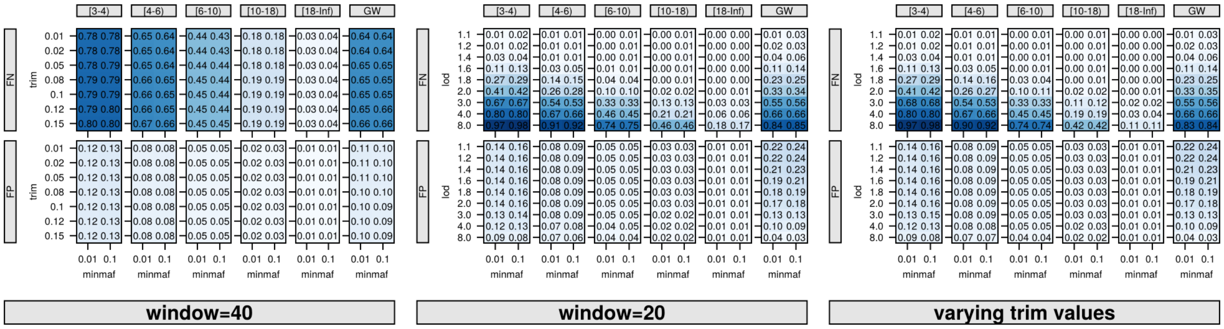

l, `Refined IBD`, multiple population model, *Pf* recombination rate.

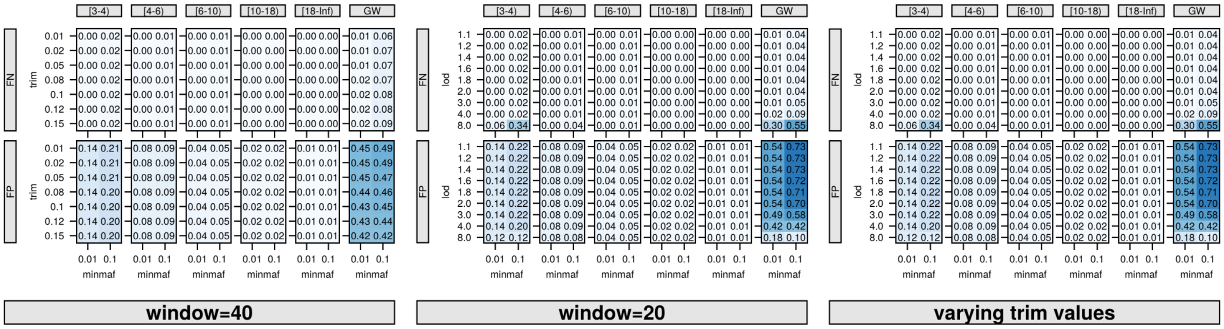

m, `Refined IBD`, UK human population model, Human recombination rate.

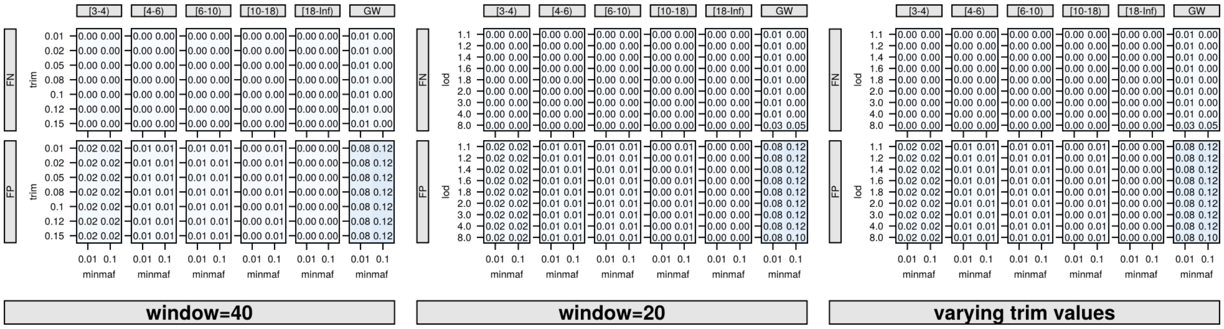

n, `Refined IBD`, UK human population model, *Pf* recombination rate.

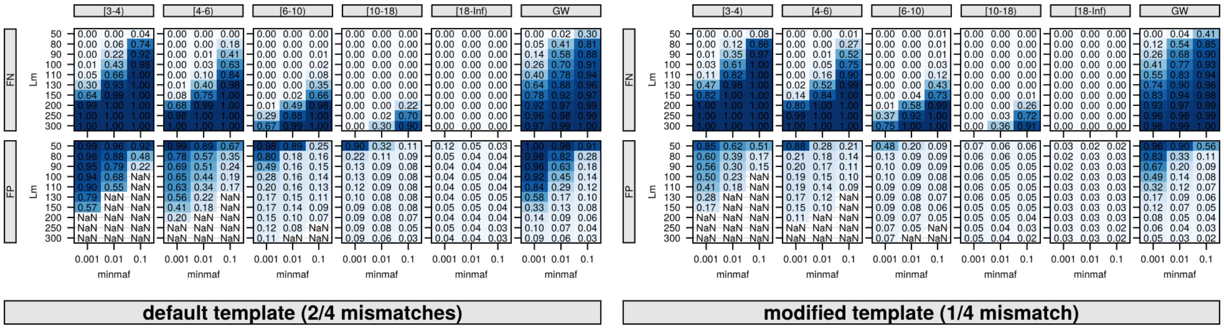

o, `phased IBD`, single population model, *Pf* recombination rate.

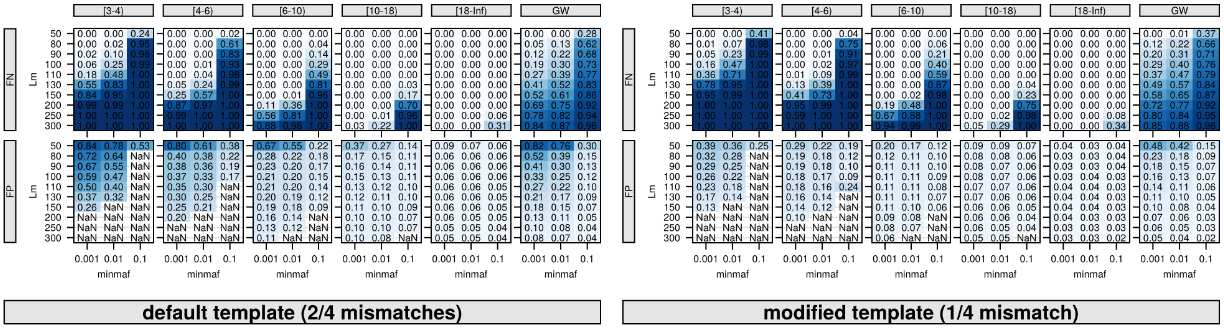

p, `phased IBD`, multiple population model, *Pf* recombination rate.

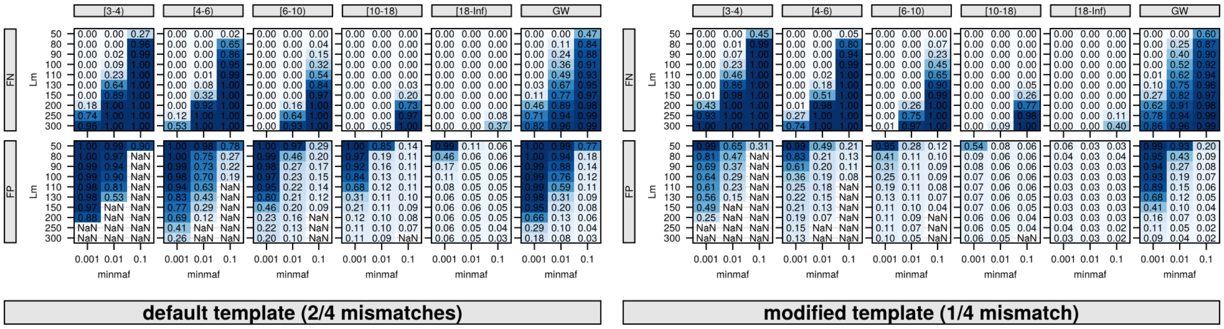

q, `phased IBD`, UK human population model, Human recombination rate.

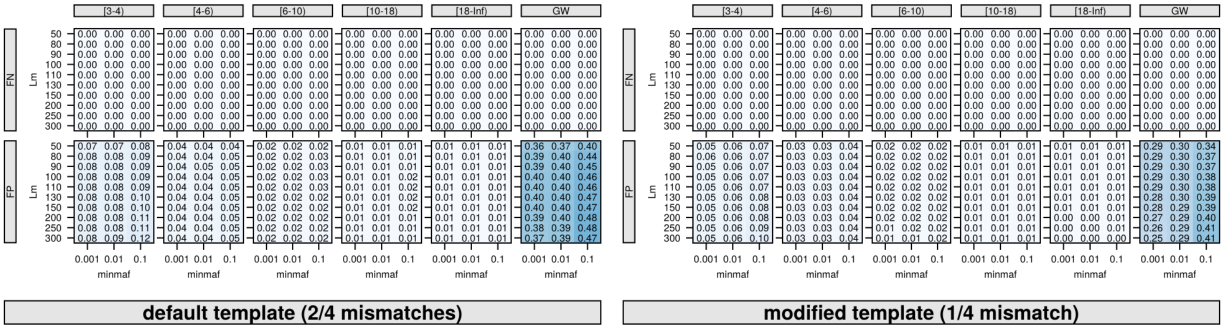

r, `phased IBD`, UK human population model, *Pf* recombination rate.

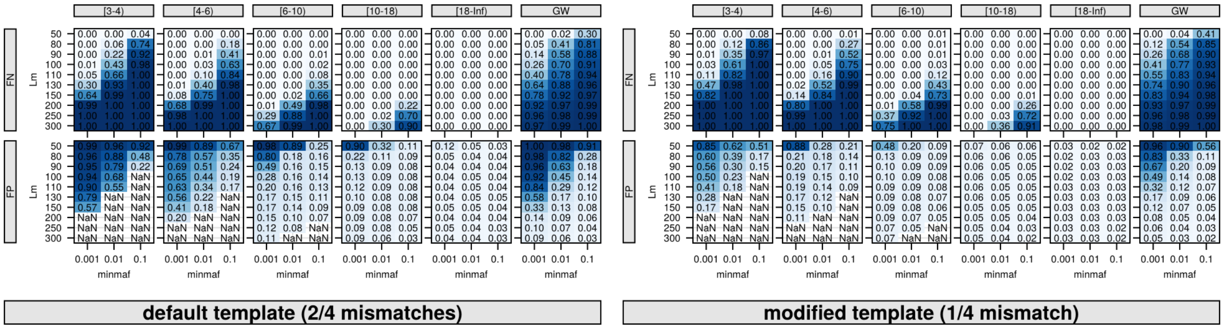

Supplement: Supplementary file 2. — (Table S1) The default and optimized values for parameters used in inferring IBD segments via different callers. A link to the source code and a citation of the corresponding article are provided for each IBD caller. For hap-IBD, Refined IBD, and hmmIBD, the parameters are used on the command line except that the parameter rec_rate of hmmIBD needs to be specified in the source code file hmmIBD.c. For phased IBD and isoRelate, the parameters are specified within a Python or R script. The details of how the parameters are specified can be found in the scripts on GitHub (https://github.com/bguo068/bmibdcaller_simulations/tree/main/bin). Note that mincm and minmaf are values shared across IBD callers to allow fair comparisons. (Table S2) Grid/Line search to optimize IBD caller-specific parameters. Column 2 lists the optimized parameters (other parameters are not explored). Column 3 shows the tested values for each parameter. Some parameters are optimized via two steps, such as coarse search (coarse) and fine-tuning (fine-tune). Column 4 provides comments on the impact of the values on IBD accuracy (measured as false positive rates (FP) and false negative rates (FN)). (Table S3) Isolates in the ‘multi-population’ data set used in empirical validation. Rows are counts of isolates from different locations (‘Population’ labels from MalariaGEN Pf7 meta information table). (Table S4) Isolates in the ‘single-population’ data set for ‘AS-SE-E’ population used in empirical validation. Rows are counts of isolates from different locations (‘Country’ labels from MalariaGEN Pf7 meta information table); columns are counts of isolates collected in a given year. (Table S5) Isolates in the ‘single-population’ data set for ‘AF-W-Ghana’ population used in empirical validation. Rows are counts of isolates from different locations (‘admin level 1’ labels from MalariaGEN Pf7 meta information table); columns are counts of isolates collected in a given year. (Data S2) Detailed IBD-level benchm [file elife-101924-supp2.pdf]
